# Diagnostic Dilemma of a Rare, Giant Retroperitoneal Schwannoma: A Case Report and Review of Literature

**DOI:** 10.1155/2014/628538

**Published:** 2014-08-27

**Authors:** Mahendra Singh, Lovekesh Kumar, Rajkumar Chejara, Om Prakash Prasad, Yuvraj Kolhe, Ashish Saxena

**Affiliations:** ^1^Department of Surgery, AIIMS, Basni, Jodhpur, Rajasthan 342001, India; ^2^Department of Surgery, Hindu Rao Hospital, New Delhi, India; ^3^Department of Surgery, VMMC & SJ Hospital, New Delhi, India

## Abstract

Schwannoma is a benign tumour of peripheral nerve sheath. It usually arises from head, neck, and trunk. Retroperitoneal schwannoma is a rare entity, accounting for only 0.3–3% of total schwannomas. Majority of retroperitoneal schwannomas reported in literature have a diameter of 5 to 15 cm. Preoperative diagnosis is difficult due to low frequency, nonspecific clinical course, and nonspecific imaging features. Histology usually provides definitive diagnosis. Schwannomas are solitary, well-circumscribed, and noninvasive, so complete surgical excision provides good result. We report a case of a 23-year-old male, who presented with progressive abdominal distension and intermittent episodes of intestinal obstruction. CECT was suggestive of huge solid-cystic mass in abdominopelvic region. Image guided percutaneous aspiration revealed around 1 litre of frank pus and FNAC was suggestive of abscess. Exploratory laparotomy revealed a large 32 × 28 × 26 cm mass with solid and cystic components containing 1 litre of pus. Histological features of tumour were suggestive of benign schwannoma and immunohistochemistry for S-100 was positive. Postoperative recovery was uneventful. We report this case of a retroperitoneal schwannoma because of giant size, rare location, unusual presentation, and diagnostic dilemma.

## 1. Introduction

Schwannoma is a benign, slow growing tumour originating from the Schwann cell. Majority of them arise from cranial nerves or nerves of upper extremities [1]. We report a rare case of giant retroperitoneal schwannoma measuring 32 × 28 × 26 cm, which was difficult to diagnose preoperatively.

## 2. Case Report

A 23-year-old male presented with history of abdominal distension since one year, which was insidious in onset and rapidly progressive. There was history of intermittent episodes of intestinal obstruction for the last 6 months. The patient was cachectic and febrile and was having tachycardia. There were no features suggestive of Von Recklinghausen's disease. Abdominal examination revealed an enormous rounded mass, which filled the entire abdomen. It was immobile, nontender, and variegated in consistency. Boundaries of mass were indistinct as superiorly it was extending below costal margins and inferiorly into the pelvis.

**Figure 1:**
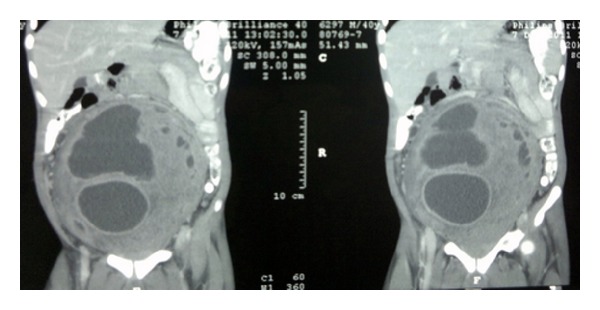
CECT (sagittal section) showing large solid-cystic mass occupying abdominopelvic region.

**Figure 2:**
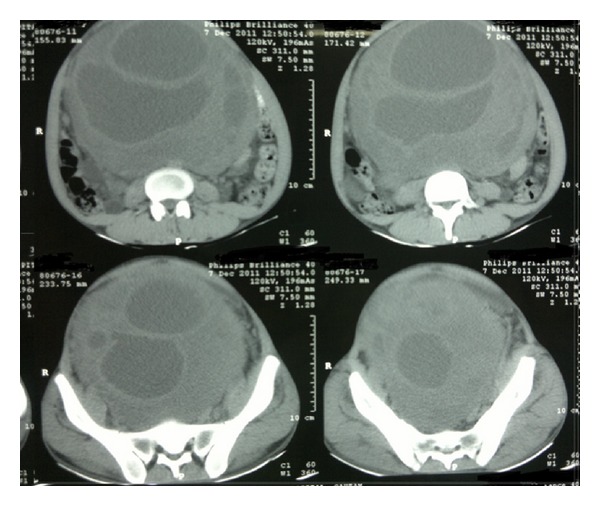
CECT showing large solid-cystic mass arising from sacrococcygeal region.

Routine blood investigations were within normal range except haemoglobin (6 gm%). CECT (contrast enhanced computed tomography) features were suggestive of a large, lobulated, relatively well-defined, heterogeneously enhancing solid-cystic mass in abdominopelvic region (Figure 1). Mass was extending up to the margin of pancreas in superior direction and inferiorly it was arising from rectovesical pouch displacing bladder anteriorly and rectum posterolaterally (Figure 2). Anterolaterally, it was reaching up to the abdominal wall with displacement of bowel loops laterally. Posteriorly it was compressing IVC, aorta and its branches, and common iliac artery (Figure 2). There was right moderate hydronephrosis with delayed excretion of contrast compared to left kidney. Image guided FNAC revealed frank pus and was suggestive of an abscess. Around one litre of frank pus was aspirated using image guided percutaneous aspiration. Culture for acid fast bacilli was negative.

**Figure 3:**
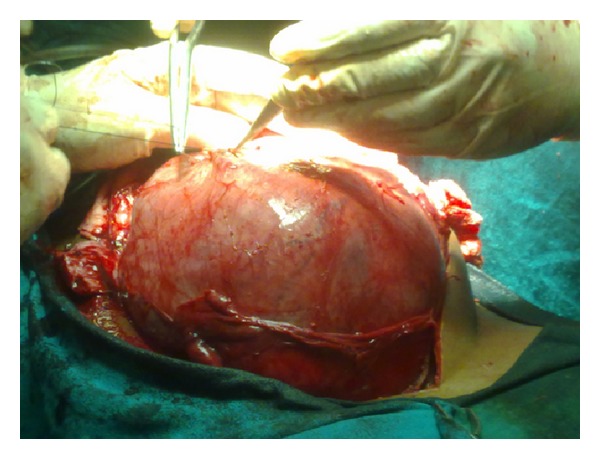
Intraoperative photograph showing giant tumour with right ureter passing anteriorly.

**Figure 4:**
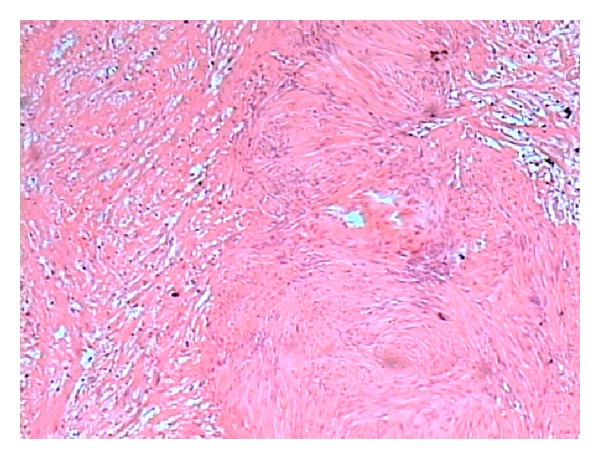
Histology of schwannoma showing well-organised spindle cells in a palisade pattern (Antoni A) on the right and less cellular area (Antoni B) on the left.

**Figure 5:**
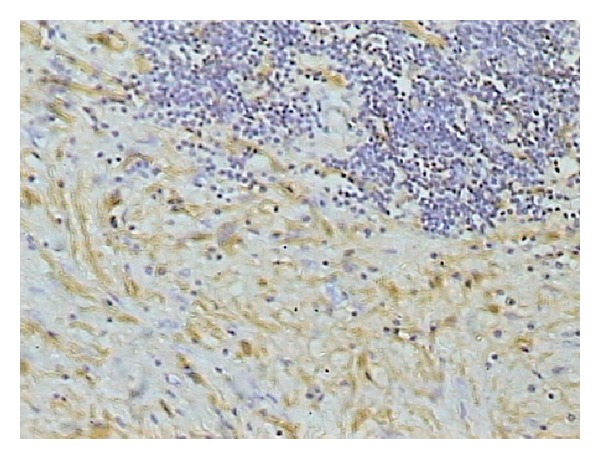
Immunohistochemistry of tumour showing S-100 positivity.

Exploratory laparotomy through midline revealed a large 32 × 28 × 26 cm mass with solid and cystic components occupying whole abdomen. The mass contained around one litre of pus. Bowel loops were pushed to left upper quadrant of abdomen. Proximal right ureter was dilated and distal part was collapsed and displaced anteriorly over the mass (Figure 3). Pelvic part of the mass was adherent to urinary bladder and rectum. On further dissection mass narrowed down and could be seen arising from sacrococcygeal region. It was impossible to trace the originating nerve; hence, the complete specimen was retrieved by sharp dissection avoiding any injury. Multiple sections examined from the mass showed histological features suggestive of benign schwannoma with areas of haemorrhage and cystic changes with focal collection of foamy macrophages (Figure 4). Fibrocollagenous tissues with chronic inflammatory infiltrates were also present. Occasional mitotic figures were present. Immunohistochemistry was positive for S-100 (Figure 5).

Postoperative recovery was uneventful. Patient is doing well and no recurrence or sensory neurological deficits were noted even after 1 year of follow-up.

## 3. Discussion

Benign schwannomas are usually slow growing tumours and predominantly occur in females (male/female ratio of 2 : 3) between the 3rd and 5th decades of life [2]. Most of schwannomas reported in literature have a diameter of 5 to 15 cm, and in our case it was 32 × 28 × 26 cm [3–5].

We found only 3 case reports of tumour of this size after extensive search. Foote et al. reported a giant retroperitoneal schwannoma of 43 × 40 × 20 cm in 1963 [3]. Schindler and Dixon reported schwannoma of 35 × 25 cm in 2002 [4]. Kuriakose et al. reported a giant retroperitoneal schwannoma (42 cm × 16 cm × 16 cm) in a 19-year-old lady [5]. Usually, benign schwannoma is a slow growing tumour, but in this case it showed rapid progression within a span of one year.

The symptoms due to benign retroperitoneal schwannomas are nonspecific and are usually associated with compression of adjacent structures [6]. The most common symptom is abdominal distension with dull abdominal pain [4, 6]. In our case, the main symptoms were abdominal distension and vague abdominal pain. Schwannoma has been associated with Von Recklinghausen's disease. Von Recklinghausen's disease is characterized by the presence of cafe-au-lait spots and multiple neurofibromas and hamartomas [6]. None of the stigmata of this disease was present in our case.

CECT and magnetic resonance imaging (MRI) are widely used as imaging methods for evaluation of retroperitoneal soft tissue tumours. MRI is usually regarded as the imaging modality of choice for most soft tissue lesions. On CECT, schwannoma appears as a well-defined, homogeneous mass with rim enhancement of the fibrous capsule [7]. The degenerative histological features of schwannomas appear as well-circumscribed complex cystic masses with inhomogeneous contrast enhancement. Nonenhancing areas on CECT correspond to regions of cystic degeneration, with contrast enhancement seen in surrounding tissues [7]. The diagnostic value of CT appears to be mitigated by its limited resolution of soft tissue. CT images fail to adequately reproduce stroma heterogeneities of schwannomas, when compared to MRI. MRI using gadolinium contrast has been advocated as superior to CT in demonstrating tumour cystic degeneration, defining margins, and in some cases identifying the point of neuronal origin. Schwannomas characteristically show low signal intensity on T1-WI similar to muscle and high signal on T2-WI similar to fat [7, 8]. These findings are characteristic but not specific of schwannomas and reported to be present in only 57% of the cases in previous studies. MRI is also incapable of reliably distinguishing between benign and malignant schwannomas [8]. The differential diagnosis for schwannoma is neurofibroma, paraganglioma, pheochromocytoma, liposarcoma, malignant fibrous histiocytoma, and hematoma [9]. In our case, aspiration of around 1 litre of pus from tumour and presence of fever and tachycardia were increasing our difficulties to make a diagnosis of schwannoma. Yet, imaging is helpful in therapeutic planning by giving information about the tumor's size, location, and possible invasion of other structures [7, 8]. Heterogeneity and cystic changes in schwannomas, as seen on CT scan, have been reported as signs of malignancy. Malignant schwannomas may also show irregular contour and invasion to surrounding structures on imaging [8]. In our case, tumour appeared well-defined without any irregularity or invasion to surrounding structure. Tumor showed heterogeneity and cystic degeneration, but on histology it was confirmed to be benign. Image guided biopsy and fine needle aspiration are not sufficient for the diagnosis as degenerated areas can hinder the correct diagnosis [8, 9]. FNA in our case was suggestive of abscess.

Definitive diagnosis is based on histological analysis of biopsy specimens. Histological studies show different structural areas identified as Antoni A and Antoni B. The A areas express a solid hypercellularity and the B areas are hypocellular [9]. IHC studies absolutely confirm diagnosis through S-100 protein positivity [9]. In our case, tumour showed the above-mentioned histological features and S-100 positivity.

Schwannomas are well-circumscribed, firm, smooth-surfaced tumours. Treatment of choice is local excision as they are noninvasive. After complete excision, tumour does not recur so adjuvant therapy is not required. Identification of the originating nerve is not always possible and a minor degree of neurological impairment may be present afterward [10]. In our case, the patient is doing well and no neurological deficit is noted even after one year of follow-up.
